# A Bioactive Emulgel Formulation of *Equisetum telmateia* Ehrh. Methanol Extract: Integrating Antioxidant Activity, Skin Enzyme Inhibition, and Permeation Kinetics

**DOI:** 10.3390/gels11080662

**Published:** 2025-08-20

**Authors:** Tuğba Buse Şentürk, Timur Hakan Barak, Emre Şefik Çağlar, Emine Saldamlı, Ebru Özdemir Nath, Zafer Ömer Özdemir

**Affiliations:** 1 Department of Pharmacognosy, Faculty of Pharmacy, Acıbadem Mehmet Ali Aydınlar University, Ataşehir, 34752 İstanbul, Turkey; tugba.avci@acibadem.edu.tr; 2 Department of Pharmacognosy, Hamidiye Faculty of Pharmacy, University of Health Sciences, 34688 İstanbul, Turkey; 3 Department of Pharmaceutical Biotechnology, Hamidiye Faculty of Pharmacy, University of Health Sciences, 34668 İstanbul, Turkey; s.emrecaglar@gmail.com; 4 Department of Pharmaceutical Technology, Faculty of Pharmacy, İstanbul University, 34116 İstanbul, Turkey; eminesaldamli@istanbul.edu.tr; 5 Natural Products R&D Centre, Altınbaş University, 34144 İstanbul, Turkey; ebru.ozdemir@altinbas.edu.tr; 6 Department of Analytical Chemistry, Hamidiye Faculty of Pharmacy, University of Health Sciences, 34688 İstanbul, Turkey; zaferomer.ozdemir@sbu.edu.tr

**Keywords:** *Equisetum telmateia*, skin-related enzyme inhibition, LC-MS/MS, emulgel, HPLC

## Abstract

*Equisetum telmateia* Ehrh. (great horsetail) belongs to the Equisetaceae family and its aerial parts have been traditionally used for skin conditions and to achieve healthy and resilient skin, nails, and hair. This study aimed to evaluate the inhibition of skin-related enzymes by, the antioxidant capacity of, and the phytochemical composition of *E. telmateia*. Additionally, a novel emulgel was formulated from the main methanolic extract and characterized in terms of pH, viscosity, determination of content quantification, textural profile analysis, and spreadability. After the characterization studies, in vitro release and ex vivo permeation and penetration studies were performed. Firstly, the dried aerial parts of *E. telmateia* were macerated in methanol, followed by partitioning with solvents of increasing polarity: *n*-hexane, chloroform, ethyl acetate, and *n*-butanol. Antioxidant activity was assessed using DPPH, FRAP, CUPRAC, and TOAC assays, while enzyme inhibition was analyzed for collagenase, elastase, hyaluronidase, and tyrosinase. LC-MS/MS analysis identified 53 phytochemical compounds. Protocatechuic acid, the main phenolic compound, was quantitatively analyzed in each subfraction by HPTLC. The in vitro release studies showed sustained release of the reference substance (protocatechuic acid) and the kinetic modeling of the release was fitted to the Higuchi model. The ex vivo permeation and penetration studies showed that the formulation exhibited a retention of 3.06 ± 0.21 µg.cm^−2^ after 24 h, whereas the suspended extract demonstrated a skin retention of 1.28 ± 0.47 µg.cm^−2^. Both the extracts and the formulated emulgel exhibited inhibitory effects on skin-related enzymes. Our finding suggested that *E. telmateia* might be a valuable ingredient for wrinkle care and skin-regenerating cosmetics.

## 1. Introduction

For centuries, medicinal plants have been applied topically to aid in skin regeneration and treat dermatological issues, including chronic diabetic wounds, acne, skin aging, burns, and persistent wounds [1]. Many natural plant-based agents support natural repair processes of the skin, demonstrating significant therapeutic potential for regeneration and wound healing through a variety of mechanisms. Based on the available literature, much of the research has concentrated on the antibacterial and antifungal effects of plants traditionally used for treating skin disorders, leaving a considerable gap in studies exploring other biological activities of these medicinal plants [2].

The skin, the body’s most visible organ, is made up of three layers: the epidermis, dermis, and hypodermis and acts as the main barrier between the body and the external environment [3]. The extracellular matrix (ECM) is the largest component of the dermis, forming a three-dimensional structure that supports growth and flexibility by providing a structural framework. Recent studies suggest that the ECM not only provides structural support to cells but is also essential for skin development and elasticity and has a key role in skin regeneration [3,4]. The ECM consists of structural proteins such as collagen and elastin, as well as fibroblasts, which are specialized cells responsible for synthesizing and maintaining these matrix components [5]. Collagen is an essential protein found in the extracellular spaces of various animal connective tissues, accounting for approximately 25–35% of the entire protein content in the body. Various collagen types and structures are of crucial importance to provide mechanical stability, flexibility, and strength to tissues and organs [6,7]. Elastin is an ECM protein that makes up approximately 2% to 4% of the dermis matrix. Despite this relatively low rate, it mediates important structural, mechanical and cell signaling roles. It is especially critical for the flexibility, quality, and durability of many vertebrate tissues [8]. The moisture of the skin is retained by hyaluronic acid and it is involved in rapid tissue proliferation, regeneration, and repair [4]. Collagenase, elastase, and hyaluronidase are responsible for the degradation of collagen, elastin, and hyaluronic acid, respectively. Therefore, degradation of the ECM components are linked to increased activity of these enzymes. These enzymes play an important role in remodeling and repair of tissue, including regulating the breakdown and deposition of the extracellular matrix [9]. For this reason, the identification and targeting of these enzymes are considerably important for the development of products in the pharmaceutical and cosmetic industries [10].

Exposure to UV radiation, certain chemicals, and smoking induces the production of reactive oxygen species (ROS), leading to oxidative stress that breaks down the ECM components. Furthermore, the activation of tyrosinase triggered by UV exposure or ROS leads to excessive melanin production, causing skin pigmentation disorders [11]. Antioxidant agents provides great potential to defend ROS and reduce oxidative stress. Therefore, a substance with potent antioxidant activity may help to protect the skin from oxidative damage and slows down the aging process [12].

In contrary to adverse effects of the synthetic materials, the use of herbal components has emerged as a safe and promising alternative to treat skin conditions such as aging, wound, and skin regeneration [13]. The formulation of skincare products often uses herbal compounds in particular due to their antibacterial, antioxidant, and skin-soothing/moisturizing/protective properties [14]. Among the benefits of emulgel over traditional topical treatments are its thixotropic properties, greaselessness, superior spreadibility, ease of removal, biodegradability, emollience, convenience, and also the improved solubility of hydrophilic and lipophilic molecules, since herbal extracts contains both. Additionally, the exceptional stability and long shelf life of emulgels encourage researchers to develop new products [15]. Previous studies on plant-based emulgels have reported that emulgels are suitable and advantageous for the formulation of cosmeceutical and nutraceutical products [16]. In addition to all these aspects, many infrastructures need to be changed, especially in order for modern drug delivery systems (nano-based drug delivery systems) to be produced in the facilities of pharmaceutical industry. Today, there are emulsion and gel production areas in the existing facilities. This makes emulgel production cost-effective. Unlike traditional drug production, emulgels are still seen as an emerging field. For all these reasons, emulgels can be easily adapted to the existing infrastructure of pharmaceutical companies and will increase the solubility of both hydrophilic and lipophilic substances, especially in herbal extracts, and thus were preferred for this study.

The genus *Equisetum* (Equisetaceae), comprising 41 identified species, has a near-global distribution [17]. Traditional records have revealed that *Equisetum* species were used as medicinal plant to treat acne, boils, chronic eczema, and wounds [18,19,20]. In addition, *Equisetum* plants are used for strong hair, nails, and skin owing to their high silica content [21]. *Equisetum telmateia* (ET), known as great horsetail, is the largest member of the Equisetaceae family and its aerial parts has been used in folk medicine to heal wounds [22]. Scientific investigations have reported that different parts of the plant possess various pharmacological activities such as antimicrobial, antiulcerogenic, antioxidant, anticholinesterase, and antiurease effects. These activities of horsetail are associated with the presence of various classes of secondary metabolites, including minerals, phytosterols, alkaloids, and phenolics [23]. As a result, ET has the potential to be a valuable source of biologically active compounds.

In the current study, the in vitro inhibitory effect of ET on skin-related enzymes was investigated to understand its potential use for skin conditions. Thus, the present study may support the reported traditional uses of ET with scientific studies. The anti-oxidant capacity of the extracts were evaluated by DPPH, FRAP, CUPRAC, and TOAC assays. Additionally, numerous bioactive substances was screened to elucidate phytochemical characterization of ET via LC-MS/MS. Then, all extracts were qualitatively and quantitatively analyzed by HPTLC to determine the possible contribution of the major phenolic compound on the biological activity. Moreover, a novel emulgel was formulated from the main methanolic extract and characterized in terms of pH, viscosity, determination of content quantification, textural profile analysis, and spreadability. After the characterization studies, in vitro release and ex vivo permeation and penetration studies were performed. The results show that ET can be a potential agent for skin conditions.

## 2. Results and Discussion

### 2.1. Phytochemical Evaluation

Generally, the secondary metabolites that plants possess are responsible for their unique biological functions. Therefore, elucidating the phytochemical profiles of plants is crucial for identifying the compounds responsible for their effects.

**Figure 1:**
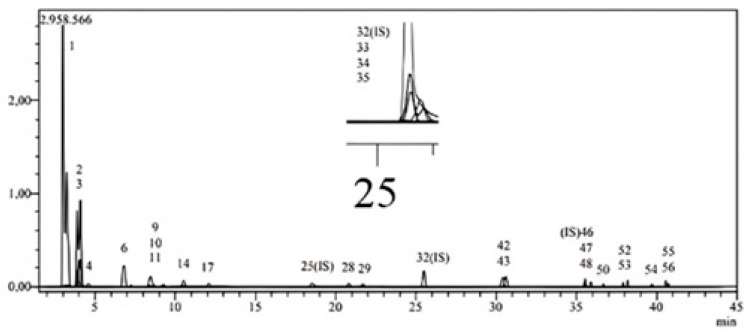
LC-MS/MS chromatograms of ETM.

The objective of this study was to clarify the phytochemical profile of ET. First, an LC-MS/MS system was used for quantitative analysis of 53 phenolic components for ETM. Table 1 displays the results and analytical parameters of this analysis. The chromatograms of the standard compounds and the extract are given in Figure 1.

In our study, the major phenolic compound in ETM was detected as protocatechuic acid by LC-MS/MS (8.03 μg/g). It was followed by equisetic acid with 7.865 μg/g, kaempferol 3-O-rutinoside with 5.334 μg/g, and kaempferol 3-O-glucoside with 4.764 μg/g. Considering the previous studies conducted to elucidate the phytochemical profile of ET, Yeganegi et al. identified kaempferol 3-O-(6″-O-acetylglucoside) as the main compound in the extract of ET obtained by SFE by HPLC analysis [22]. Additionally, phenolic acids such as protocatechuic acid (2.4%), p-hydroxy-benzoic acid (4.5%), 5-O-caffeoyl shikimic acid (15.2%), and monocaffeoyl meso-tartaric acid (1.3%) acids have been detected.

In another study, analysis of the ethyl acetate fraction obtained from the infusion of ET by HPLC-PAD-ESI/MS showed that the main flavonoids in this fraction were kaempferol derivatives and flavan-3-ols, and the method also allowed the identification of protocatechuic acid and p-hydroxybenzoic acids [24]. Also, Nunes et al. reported that only protocatechuic acid was detected in ET by RP-HPLC [25]. Reviewing the literature studies conducted on other *Equisetum* species, kamferol 3-*O*-glucoside has been reported in *Equisetum arvense* L. and *Equisetum giganteum* L., and kamferol 3-*O*-glucoside and kamferol 3-*O*-rutinoside have been reported in *Equisetum* palustre L., again consistent with our findings [26,27,28]. In another study using UHPLC-DAD, ET showed the highest chlorogenic acid content among *Equisetum* species, with 10.12 mg/g in methanol and 14.38 mg/g in ethanol extracts [29]. However, in our study, chlorogenic acid (0.013 μg/g extract) was detected in a very low amount in the methanol extract. Similarly, Roumili et al. were unable to detect chlorogenic acid in the methanol extract of *Equisetum arvense* L. using HPLC [30]. This variation is dependent upon the plant’s chemical content profiles, which are influenced by the extraction solvent as well as environmental factors such as temperature, humidity, soil quality, and availability of water. It is also affected by harvesting time, storage conditions, and extraction technique.

**Figure 2:**
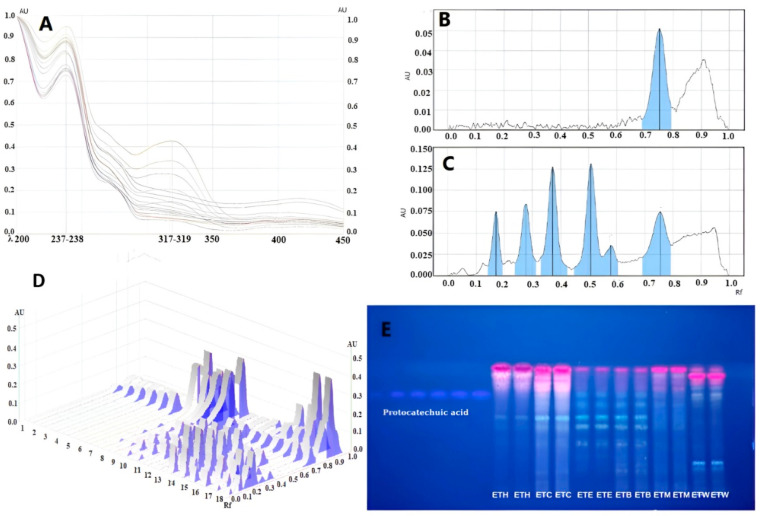
HPTLC analysis of the extracts. (**A**) UV spectra of protocatechuic acid and same Rf value of tracks at 200–450 nm. (**B**) HPTLC chromatograms of protocatechuic acid. (**C**) Example chromatogram of ETE (ethyl acetate extract of ET). (**D**) 3D HPTLC chromatogram of protocatechuic acid analysis of Equisetum telmateia extracts at 366 nm. (**E**) HPTLC chromatograms of ETH: hexane fraction of ET, ETC: chloroform fraction of ET, ETE: ethyl acetate fraction of ET, ETB: n-butanol fraction of ET, ETM: methanolic extract of ET, ETW: remaining water fraction of ET. Mobile phase: toluene: ethyl acetate: formic acid (7:5:0.5). Derivatization: NPR reagent. Visualization: 366 nm.

Later, we aimed to determine the distribution differences between the fractions by HPTLC of the major phenolic compound detected in the main methanol extracts by LC-MS/MS. The main reason for the investigation of the key phenolic compound is to determine its possible contribution to the activities in the fractions. In recent years, with the increasing understanding of their biological activity levels, phenolic compounds have been found to exhibit various effects such as antioxidant, antimicrobial, antiproliferative, and anti-inflammatory effects and prevention of cardiovascular diseases, cancers, diabetes, and oxidative stress-related diseases [31]. Given this information, it can be suggested that phenolic metabolites may play crucial roles in the biological activities of plants. The results of the HPTLC are given in Table 2 and Figure 2.

In the LC-MS analysis of ETM, the major bioactive compound was identified as protocatechuic acid. Protocatechuic acid (PCA), chemically identified as 3,4-hydroxybenzoic acid, is a prominent secondary metabolite belonging to the phenolic acid group and is commonly found in plant sources like fruits and vegetables. Earlier research has examined its anti-aging, antioxidant, and skin-whitening properties [32,33]. In light of this information, also determining the qualitative and quantitative differences in all extracts and formulations can be useful to evaluate the results of later biological activity studies.

PCA was detected at the highest rate in ETE, followed by ETB and ETM, respectively. PCA was not detected in ETH, ETC, and ETW. This is due to the fact that bioactive compounds like phenolic acids and flavonoids have a moderate polarity, allowing them to be effectively extracted using ethyl acetate, which also has mild polarity [34]. In a previous study by Nguyen et al., *n*-hexane, ethyl acetate, and methanol extracts obtained from the aerial parts of *Equisetum diffusum* D.Don were profiled using HPTLC in terms of flavonoids, polyphenols, and terpenoids, using specific reagents. All of these metabolite classes were present in the methanol extract, while the ethyl acetate extract included some polyphenols and flavonoids, but the n-hexane extract contained none of the distinctive class of chemicals [35]. In a study comparing the amount of PCA in different extracts of *E. arvense* L. using HPLC, consistent with our findings, ethyl acetate extract was found to be the extract containing the highest amount of protocatechuic acid (1.09 ± 0.05 mg/g), followed by *n*-butanol (0.55 ± 0.03 mg/g) and water extract (0.36 ± 0.02 mg/g) [23].

### 2.2. Preparation and Characterization of Emulgel Formulation

Today, consumers are increasingly drawn to skincare products and cosmetics made from natural ingredients. This trend has prompted many cosmetic brands to introduce more products containing plant extracts known for their anti-aging, anti-wrinkle, and skin-whitening properties, along with other benefits [36]. These plant-based substances are often used in topical skincare treatments and can address various skin issues. However, most of these claims lack scientific validation [14]. As a result, the development of herbal-based formulations with scientifically proven benefits has become a priority in the cosmetic industry.

Topical application is ideal for skin-related treatments since the skin is the most accessible organ, making it an effective route for delivering active ingredients. Topical drug delivery offers several advantages, such as targeted application, avoiding gastrointestinal issues, and preventing metabolic complications linked to oral administration. While this method has been in use for a long time, new techniques and technologies are continually being developed to enhance patient compliance [37]. Emulgel systems incorporating natural plant extracts have gained attention as a novel and effective approach in cosmetology, particularly due to the increasing interest in plant-based phytochemicals [38]. Previous studies have shown promising results with emulgel formulations containing various herbal extracts [38,39,40].

In this study, emulgel formulations using ETM extracts were developed. Table 3 presents the in vitro characterization results, including the pH and viscosity measurements.

The pH value of the skin varies between 4.0 and 7.0. Studies have shown that skin with low pH protects its existing flora better [42]. In the results obtained, F and ETF were found to be between 5.4 ± 0.1 and 6.5 ± 0.1, respectively. The compatibility of the formulation’s pH with the natural pH of the skin is a critical factor for ensuring patient compliance. The results demonstrated that both the blank and extract-loaded formulations exhibited pH values within the physiological range of the skin. Formulations with markedly acidic or basic pH values are generally considered unsuitable for topical application, as they may cause irritation or discomfort. Furthermore, the pH levels of the developed formulations were found to be appropriate for preserving the skin’s natural microbiota, thereby supporting their suitability for dermal use. In addition to all these, it is thought that it will not cause irritation on the skin. The reason why the pH value of the F formulation is lower than ETF is due to the fact that it has Carbopol in its structure. It has been determined that the pH value increases with the addition of ETM, which is a methanol extract. Furthermore, the observed increase in pH from 5.4 ± 0.1 in the blank formulation (F) to 6.5 ± 0.1 in the extract-loaded formulation (ETF) can be attributed to multiple interrelated physicochemical mechanisms. First, the addition of 4% (*w*/*w*) of methanol-based plant extract introduces both organic solvent and phytochemical constituents, which can alter the proton activity within the gel matrix. Deleebeeck et al. (2021) demonstrated that the incorporation of ethanol into aqueous systems can lead to an elevation in apparent pH values due to changes in the solvent’s autoproteolysis constant and the overall proton solvation environment, even in unbuffered conditions [43]. Additionally, Fresno et al. (2002) reported that increasing the ethanol content in Carbopol-based hydroalcoholic gels results in a shift in the pH_50_ (the pH at which 50% of gel structure forms), thereby requiring higher pH to achieve optimal polymer neutralization and gel consistency [44]. This phenomenon is explained by the ethanol-induced reduction in polymer hydration and increased need for neutralization agents such as triethanolamine. Moreover, methanol-based plant extracts may also contain weakly basic or buffering phytochemicals—such as polyphenols, alkaloids, or flavonoids—which can further contribute to the alkalinity of the formulation. Therefore, the observed increase in pH following the addition of ETM (methanol extract) is consistent with both theoretical expectations and previous experimental findings. These results confirm that the pH shift remains within the physiological limits of the skin and does not compromise formulation compatibility or safety.

Viscosity is a crucial physical attribute of topical formulations that can impact medication release rates and affect application characteristics such as spreadability and skin sensation. When a gel’s viscosity is insufficient, it must be applied rapidly to the skin, as it tends to flow off swiftly [45]. When the results were examined, the viscosity decreased significantly with the addition of ETM. The presence of alcohol in the methanol extract led to a reduction in the ability of the remaining carboxyl groups to form hydrogen bonds, thereby decreasing the viscosity of the formulation. This outcome is consistent with the fact that aqueous gels generally exhibit higher viscosity compared to hydroalcoholic gels [46]. This facilitated the applicability of the developed formulation. This situation is compatible with the spreadability studies.

In addition to all these, in the content quantification study, it was determined that the reference substance was loaded with emulgel at a rate of 77.19 ± 2.07%. In a formulation in which a topical emulgel of metronidazole was developed, the drug content result was found to be between 70 and 90% [47].

### 2.3. Texture Profile and Spreadability Analysis

Texture profile analyses and spreadability analyses of these formulations were performed. Table 4 and Table 5 show the findings from the spreadability investigation and texture profile analysis of the emulgel formulation.

Understanding the order and interactions between the component parts of a formulation was supported by examining the mechanical textural properties of a semisolid emulsion system. By subjecting the sample to an external compressive stress and assessing the formulation ability to produce both reversible and irreversible deformations, TPA can determine these mechanical properties [48,49,50]. The TPA technique is a quick and easy way to obtain information about the physical structure of an emulsion system [51]. The mechanical textural properties of the formulations were analyzed at 25 °C. The hardness (N or g), defined as the resistance of a product to deformation, is determined by the initial maximum compressive force. It is an indication of the hardness of a gel that remains in place after application to the skin, which provides information about the ease of application of the gels. The removal of the probe from the formulation is defined by the tackiness value, which is related to the adhesive properties. An ideal property for long-term drug retention is a higher degree of tack, indicating stronger tissue surface adhesion. The tackiness for the first compression cycle is determined using a negative force field when there is an attractive force between the gel surface and the probe. The initial compression deformed the gel sample, and the gel’s regeneration after deformation is defined by its springiness or elasticity. How formulations respond to repeated shear stresses is indicated by their viscosity. Comparing the ability of a product to regroup after one deformation with its ability to regroup after another deformation allows determining how sticky the product is. When the formulations were examined (Table 4), the formulations containing extracts exhibited lower hardness, adhesiveness, and elasticity values compared to the blank formulations. The viscosity results are also consistent with these results. The primary reason for these results is the effect of environmental ion concentrations on the gel-forming chemical used. The methanol content of the extracts was found to have a gelation-reducing effect. When Zamaora and colleagues examined the mechanical properties of the plant extract-loaded antimicrobial gel they created, they found that the extract-loaded gel had lower mechanical properties than the blank gel [52]. The lower mechanical properties of the extract-loaded gel compared to the blank gel are also significant for the patient. Its rapid gelation ability and low viscosity produced a easily manageable gel. Likewise, potential adverse effects on the skin can now be more easily eliminated.

Spreadability is a key factor in topical formulations, as it significantly impacts patient compliance. A formulation with good spreadability is easier to apply and can cover more skin, enhancing its therapeutic effectiveness [53]. Spreadability testing evaluates the variation in force over time, reflecting properties such as firmness, cohesiveness, spreading work (shear work), and extrusion force (adhesion work). The formulation’s hardness, or the strongest positive force required to deform it, indicates its strength, with higher hardness reflecting a stronger formulation. The amount of shear work, shown by the area up to the positive force, measures the formulation’s spreadability. The negative portion of the force graph, influenced by the sample’s weight on the rotating probe, shows the resistance to sticking or flow during the process. The maximum negative value represents the cohesive force needed to separate the gel from its container, with the cohesive work being represented by the area under the negative curve [54]. Hardness and shear work are inversely related to spreadability. Therefore, as the hardness value increases, the spreadability of the formulation decreases, making it more difficult to apply [55]. The spreadability outcomes (Table 5) were influenced by reduced gelation capacity and the mechanical properties of the extract-loaded gels. The results demonstrated that the extract-loaded gels had superior spreadability compared to the blank formulations, aligning with the viscosity and texture profile analysis (TPA) values. In extract-loaded formulations, the hardness value tends to decrease, which is also associated with a reduction in viscosity. This indicates that the formulation can be applied more easily, supporting its suitability for topical administration.

### 2.4. In Vitro Release Studies

**Figure 3:**
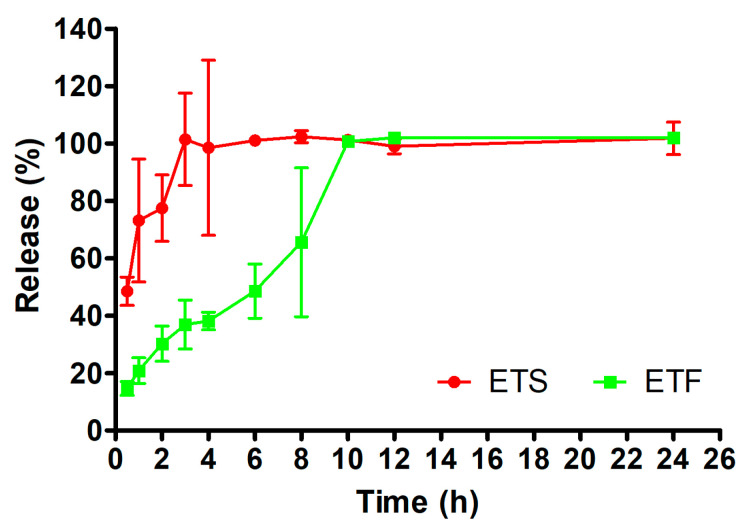
Cumulative in vitro release of protocatechuic acid from ETS and ETF.

**Figure 4:**
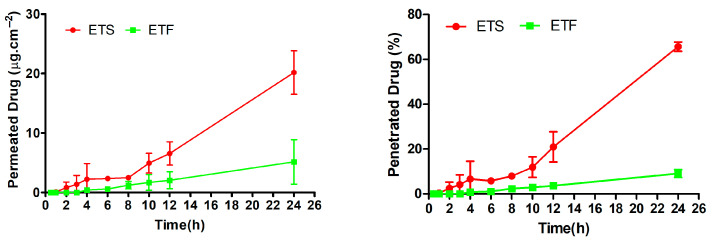
Cumulative ex vivo permeation and penetration of protocatechuic acid of ETS and ETF.

In vitro release studies of the formulations (ETS: suspended methanol extract of ET in distilled water and ETF: emulgel formulation of methanol extract of ET) were carried out using dialysis membranes, and the reference agent quantification in the release medium was determined by the HPLC method on samples taken from the release medium at certain time intervals. The results of the in vitro release study of the formulations are shown in Figure 3. Upon evaluation of the release data after 24 h, it was determined that ETF and ETS exhibited release percentages of 102.00 ± 1.00% and 101.76 ± 5.67% of the reference agent, respectively. However, when Figure 4 is examined, it is seen that ETS releases approximately half of the reference material with 48.47 ± 4.95% in the first half hour and releases the entire reference material in the third hour. On the other hand, ETF was found to release 14.66 ± 2.44% of the reference substance in the first half hour, and to release the entire reference substance at the tenth hour. According to the in vitro release results obtained, ETF showed controlled release.

#### 2.4.1. Kinetic Release Modeling

Some models may improve initial studies and produce compelling outcomes. Before carrying out linear regression analysis, the data for each instance were converted. Zero-order release kinetics often denotes a consistent release of the encapsulated substances into biological fluids [56]. Moreover, the zero-order law may be utilized to ascertain major secondary metabolite (MSM) delivery methods wherein the MSM dissolves at a constant rate, regardless of the initial drug concentration, and where the medication does not undergo increasing degradation. First-order release kinetics signifies that the variation in substance concentration over time is only contingent upon the concentration itself [57]. Initially, the Higuchi model was employed to quantify drug release from matrix systems; nevertheless, it subsequently became the favored approach for studying drug transport in various geometries and porous matrices. The Higuchi model enabled the creation of other mathematical models crucial for categorizing probable release profiles of drugs in dosage forms, including the Korsmeyer and Peppas models [58]. The Hixson–Crowell cube root equation is a mathematical model employed to describe MSM release, modified to account for the decrease in solid surface area with time [59]. The data indicated that ETS and ETF predominantly conformed to the Higuchi model (Table 6).

The in vitro release characteristics of the reference agent for these formulations were examined utilizing methods grounded in the Korsmeyer–Peppas model [60]. Consequently, this study demonstrated that ETS and ETF exhibited Fickian diffusional release processes, with “*n*” values of 2.4873 and 2.0815, respectively.

#### 2.4.2. Similarity Studies

The calculated difference and similarity factors for pair-wise intraformulation comparisons are shown in Table 7. ETS compared to ETF and was showed statistically different release profile (f1: 66, f2: 15); in addition, ETF compared to ETS were found to be significantly different (f1: 193, f2: 15).

In a conducted study, the researchers developed an ocularly applicable nanofiber and compared the in vitro release results using similarity testing. Among the obtained results, values of *f*_1_: 271 and *f*_2_: 15 indicated that the formulations exhibited different release profiles. Similarly, in the same study, *f*_1_: 73 and *f*_2_: 143 were also reported, further emphasizing the differences in the in vitro release profiles of the formulations [61].

### 2.5. Ex Vivo Permeation and Penetration Studies

Figure 4 illustrates the results of this case study, which examined MSM penetration into ex vivo skin tissue. The results indicated that ETF exhibited a retention of 3.06 ± 0.21 mg.cm^−2^ after 24 h, whereas ETS demonstrated a skin retention of 1.28 ± 0.47 mg.cm^−2^ (Table 8). Furthermore, after 24 h, the amount of the reference compound that permeated from the gel was significantly lower compared to that from the suspended extract. The ETS and ETF were assessed for the reference agent’s permeation coefficient (Kp) and steady-state flux (Jss) [61]. In comparison to ETF, ETS exhibited significantly elevated Jss and Kp values. These results indicate that the inclusion of the reference compound within the gel matrix may influence and potentially regulate the permeability characteristics of the skin tissue.

The findings demonstrated that the integration of extract into gel bases led to a delayed penetration, attributable to the introduction of an additional diffusion barrier, while enhancing skin retention (Table 9). Given that the formulation is designed for efficacy in anti-aging products, it is advantageous for the reference agent to accumulate within the skin.

The investigation revealed that the permeation and penetration findings of ETS exceeded those of the ETF formulation. The findings align with the in vitro release research. The postponed release of the MSM from the gel system also influences the ex vivo characteristics of the extract. According to the in vitro release graph (Figure 4), ETS exhibited around 50% release at 0.5 h, whereas ETF demonstrated roughly 15% release at the same time point.

Fiqri et al. conducted a study to enhance the localization of cefazoline sodium thermosensitive mucoadhesive hydrogels in ocular tissues [62]. They created an in situ gel system utilizing F127 and F68 poloxamers with hyaluronic acid as a mucoadhesive agent. The ex vivo permeation and penetration investigation revealed that the cefazoline sodium solution exhibited superior permeation and penetration results compared to the in situ gel formulation. This was linked to release kinetics [63]. The findings align with the regulated and extended release of the ETF.

In another study, ex vivo permeation testing revealed that the aqueous control solution achieved significantly greater penetration of chlorogenic acid, rutin, and quercetin into both epidermal and dermal layers compared to all semisolid formulation bases (emulsion, gel, emulgel, ointment, oleogel). This suggests that the unconstrained solution matrix enhances the dermal uptake of phenolic compounds relative to structured topical systems. This increase in permeation may be attributed to the higher thermodynamic activity of the active compounds in the solution form, which facilitates their diffusion across the skin barrier in the absence of viscosity-modifying agents or structural matrices that could hinder molecular mobility [64].

### 2.6. In Vitro Antioxidant Tests

The skin is the most visible organ and is exposed to the outer environment [32]. As a result, the skin is a major target of reactive oxygen species and oxidative stress [65]. Oxidative stress is one of the main factors contributing to skin aging; also, excessive ROS production might lead to disrupted cellular functions and causes skin diseases such as delayed wound healing, psoriasis, acne vulgaris, vitiligo, and atopic dermatitis [33]. The presence of effective antioxidants is well known in plant extracts. The biological features of plants, especially their antioxidant capacities, are acknowledged to be significantly affected by their phenolic compounds. Furthermore, antioxidant properties of plants rely on both the solvent and yield of bioactive compounds. Thus, the most appropriate solvent selection affects the extract qualities; each matrix solvent system has a unique, unpredictable behavior because of the differences in composition and structure [66]. With respect to this, antioxidant activity was evaluated using CUPRAC, FRAP, TOAC, and DPPH radical scavenging activities in the methanol extract, novel formulation, and each of the subfractions of the plant. In this way, the present study aimed to compare the effects of the solvent differences and the developed formulation on phenolic composition and antioxidant activity. The results are given in Table 8.

The largest capacity in neutralization of DPPH radicals was measured in the methanol extract from ET. Similarly, Radojevic et al. investigated antioxidant capacity methanol, ethyl acetate, and acetone extracts of ET. The results show that methanol extract of ET has the highest antioxidant activity [67]. In another study, methanol extract of ET (IC_50_ 12.92 µg/mL) showed stronger DPPH activity than water (IC_50_ 20.32 µg/mL) and ethyl acetate (IC_50_ 50.90 µg/mL) extracts [24]. Considering the research conducted on different *Equisetum* species, Necip et al. reported that the methanolic extract of *E. arvense* showed stronger DPPH radical scavenging activity than the water extract, similarly to our study [68]. In a study evaluating the antioxidant activity of *E. arvense* in a methanol, ethanol, water, and ethanol-water (4:1) mixture, Dormousoglou and colleagues reported the highest activity in the DPPH test in the methanol extract [69]. In a study conducted with the DPPH test on different extracts of *E. ramosissimum*, methanol extract showed the highest free radical scavenging ability, with an IC_50_ of 123.89 ± 0.73 µg/mL [17]. Generally, extracts prepared with alcohol showed stronger antioxidant activities.

Although PCA was not detected in the chloroform fraction, it exhibited significant antioxidant activity in the DPPH, CUPRAC, and FRAP tests. These results were equivalent to or higher than the ethyl acetate fraction with the highest PCA content. In a previous study in which the antioxidant capacity of petroleum ether, chloroform, and methanol extracts prepared from the rhizomes of *Iris suaveolens* Boiss. & Reut. was determined using β-carotene-linoleic acid and CUPRAC methods, the highest inhibitory capacity was determined in the chloroform extract by Hacıbekiroglu et al. The chloroform extract was also determined to be the richest extract in terms of phenolic compounds (437.69 ± 0.82 µg) among the tested extracts [70]. Phenolic compounds, classified as secondary metabolites, play an important role in maintaining the balance between oxidants and antioxidants during defense against oxidative stress in the human body. Among these, phenolic acids and flavonoids in particular have been the subject of numerous studies on their antioxidative activity, mainly due to their capacity to act as free radical scavengers and/or metal chelators [23]. The phenolic content of plant extracts is influenced by the selection of extraction solvent, as well as the inherent properties of the phenolic compounds, which also impact the activity of the extracts. In conclusion, no correlation was found between the antioxidant activity and amount of PCA present. It can be difficult to identify the exact components in the extracts that contribute to the antioxidant action because of their complex composition. This is further complicated by the possibility that the compounds in the extract may interact in ways that are synergistic, additive, or neutralizing [71]. The significant activity found in apolar fractions highlights the need to examine various phenolic compounds for better understanding of the phenolic content in these fractions.

### 2.7. Inhibition Potential on Skin-Related Enzymes

A wide range of chemical and physical factors can damage the skin, altering both its structure and function [72]. Prolonged exposure of human skin to UV radiation can lead to conditions such as sunburn (erythema), wrinkle development, changes in skin pigmentation like melasma, immune system suppression, inflammation, and skin cancer, and a decreased ability to heal wounds [73].

Melanin, a mixture of heterogeneous biopolymers, is primarily responsible for the pigmentation of human skin, hair, and eyes. Additionally, it defends the skin by scavenging ROS and absorbing up to 75% of UV radiation [74]. Specifically, tyrosinase is responsible for the catalysis of melanin within melanocytes and is one of the main regulatory enzymes of the skin. Tyrosinase inhibitors and antioxidants, which inhibit the formation of melanin and its autoxidation, have been developed through active research, and these compounds have been employed as therapeutic treatments for skin problems in humans, including skin whitening [75]. Collagenase is the main enzyme that contribute to the breakdown of collagen, affecting the structural integrity of the skin. Elastase plays an essential role in breaking down elastin, a protein that is vital to connective tissue. Hyaluronidase breaks down hyaluronic acid, a key component for retaining water and maintaining skin moisture, smoothness, hydration, and lubrication. Consequently, the degradation of hyaluronic acid by hyaluronidase leads to dry, sagging skin and a reduction in skin smoothness and elasticity [36]. The presence of these enzymes becomes dominant, leading to ECM degradation and changes in skin texture. The primary alterations linked to the change in skin appear are wrinkles, uneven form, pigmentation flecks, blemishes, and a notable reduction in skin flexibility [76]. Tyrosinase, elastase, collagenase, and hyaluronidase inhibitors work together to prevent skin aging, induce skin regeneration, and improve skin conditions. Despite extensive research into discovering new inhibitory molecules from both synthetic and natural sources, there remains a significant demand for new enzyme inhibitors due to the side effects or limited efficacy of existing options. Plant resources have long been researched for enzyme inhibitors associated with various skin diseases in the cosmetics and drugs industries [71]. Thus, the present investigation examined the inhibitory effect of different ET extracts and the novel emulgel formulation on enzymes associated with the skin.

**Figure 5:**
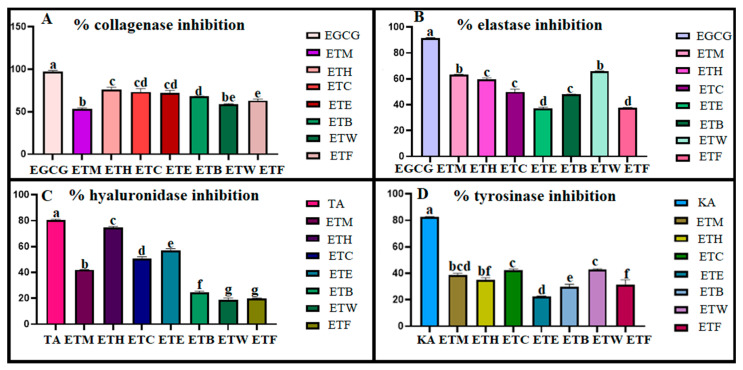
Skin-related enzyme inhibitory effect of ET, novel formulation, and all fractions. Different letters indicate significance (*p* < 0.05). Inhibitory effects of ETM (methanolic extract of ET 1 mg/1 mL), ETH (hexane fraction of ET 1 mg/1 mL), ETC (chloroform fraction of ET 1 mg/1 mL), ETE (ethyl acetate fraction of ET 1 mg/1 mL), ETB (n-butanol fraction of ET 1 mg/1 mL), ETW (remaining water fraction of ET1 mg/1 mL), ETF (emulgel formulation of ET) on collagenase (**A**), elastase (**B**), hyaluronidase (**C**), tyrosinase (**D**). Standards: EGCG: epigalloctechin gallate (1 mg/mL for anticollagenase activity, 0.25 mg/mL for antielastase activity), TA: tannic acid (1.1 mg/mL), KA: kojic acid (1 mg/1 mL).

A screening of tyrosinase, elastase, collagenase, and hyaluronidase inhibitory activity was carried out on the extracts at a fixed concentration of 1 mg/mL. The obtained results are reported in Figure 5. Considering the research on antityrosinase activity carried out on Equisetum species, the highest inhibition among ethanol, water, ethyl acetate, and 80% methanol extracts of *E. ramosissimum* was similarly shown by the 80% methanol extract (IC_50_: 1.125 mg/mL). It also showed higher activity than the kojic acid used as a standard (IC_50_: 2.132 mg/mL) [77]. In another study conducted on *E. ramosissimum*, the methanol extract prepared from the plant was fractionated as n-hexane, dichloromethane, ethyl acetate, and remaining water, respectively. When the antityrosinase activity of the samples obtained was evaluated, the ethyl acetate fraction (38.93 ± 3.09%) provided a higher inhibition percentage than standard kojic acid (30.23 ± 5.68%) [78]. In our study, all the tested samples showed lower inhibition than kojic acid. On the other hand, the ETM extract exhibited the greatest antielastase activity. In a previous study on methanol, water, chloroform, and hexane extracts of *Echinops borae* C. Vural., the antielastase activity was evaluated and the methanol (IC_50_ of 1.99 ± 0.18 µg/mL) and water extracts (IC_50_ of 2.63 ± 0.76 µg/mL) of the aerial parts showed higher inhibitory activity than the hexane (IC_50_ of 5.82 ± 1.67 µg/mL) and chloroform (IC_50_ of 8.76 ± 1.26 µg/mL) extracts [79].

Considering the anticollagenase and antihyaluronidase activities, the results show that apolar fractions have the highest inhibition capacity. The same results were also reported for *Leucas aspera* (Willd.) Linn., in a study conducted on different extracts of the plant, such as ethyl acetate, acetone, hexane, and ethanol. Since collagenase is a zinc-containing enzyme, phenolic compounds such as flavonoids, phenolic acids, phenolic diterpenes, tannins, and tocopherols have been reported to inhibit this metalloproteinase [80]. It is important to determine the chemical profiles of the apolar fractions of plants.

### 2.8. Stability Studies

Formulations were prepared and evaluated for 12 months in a stability cabinet at 4 ± 2 °C (in the refrigerator), 25 ± 2 °C at 60 ± 5% relative humidity, and 40 ± 2 °C at 65 ± 5% relative humidity. ETF was evaluated in terms of pH, viscosity, and content quantification for 12 months.

When evaluated in terms of pH and viscosity, ETF did not show significant changes at each temperature (Table 10). On the other hand, the formulation retained 92.63 ± 8.44% of the drug content when stored at 4 ± 2 °C (Table 9). Therefore, it is suggested that the developed formulation needs to be kept in a refrigerator.

## 3. Conclusions

Plant products, such as extracts, are widely used to treat and care for skin conditions and contribute significantly to maintaining and restoring the homeostasis of the skin’s protective barrier. Therefore, plants represent highly valuable resources in the development of skincare cosmetics. In this study, different extracts obtained from the aerial parts of *E. telmateia*, along with a novel emulgel formulation developed using its methanol extract, were evaluated for elastase, hyaluronidase, collagenase, and tyrosinase inhibitory activities, as well as antioxidant potential. Their effectiveness as anti-aging, wound-healing, and skin-whitening agents was assessed. The findings indicate that the developed formulations exhibit promising bioactivity profiles and hold potential for use in dermocosmetic applications. In summary, this study not only presents a comprehensive phytochemical characterization of *E. telmateia* but also introduces a novel plant-based emulgel formulation as a multifunctional topical delivery system. The innovation lies in the integration of a bioactive-rich extract into an emulgel matrix, offering enhanced skin permeability, stability, and consumer-friendly texture—features highly valued in modern cosmetic product development. This work contributes to the field by demonstrating the practical applicability of *E. telmateia* in the formulation of natural, effective, and sustainable skincare products, and provides a foundation for future studies focusing on plant-derived topical systems.

## 4. Materials and Methods

### 4.1. Chemicals

All chemicals, reagents, enzymes, and references were analytical grade or purer.

### 4.2. Plant Material

ET was collected from Helvacalı Village, Çarşamba, Samsun, Türkiye in 2019. The plant samples were described botanically by Dr. Ebru Özdemir Nath. A voucher specimen of the plant was kept in the Pharmacy Herbarium at Altınbaş University in Istanbul, Turkey (HERA 264).

### 4.3. Preparation of the Extract

Before extraction, air-dried plant materials were ground into fine powder. Then, the materials were left to macerate with a mixture of methanol/water (4:1) in a dark ambient at room temperature for 3 days (4 h in an orbital shaker). This process was repeated twice, the macerate was filtered through double-layer filter paper, and the resulting filtrates were combined and placed in tared balloons and evaporated to dryness in a rotavapor. Then, they were frozen at −80 °C and lyophilized to remove water. The total extraction yield was calculated as 5.05%. Then, the extracts were mixed with a sufficient amount of water and subjected to liquid–liquid extraction in the separating funnel with solvents of different polarities, from the most non-polar to the polar. After the solvents of the obtained fractions were evaporated in a rotavapor, yield calculations were completed and the solvents were stored at −20 °C. Finally, the main extract and 5 subfractions were obtained from the plant, including methanol, n-hexane, chloroform, ethyl acetate, n-butanol, and remaining water.

### 4.4. Phytochemical Investigation

#### 4.4.1. Determination of Phenolic Compounds by LC-MS/MS

A total of 53 phytochemical compounds in the methanolic extract of ET were identified and quantified using LC-MS/MS, following a method previously validated by Yılmaz [81]. For the quantitative analysis, a Nexera model UHPLC (Shimadzu, Kyoto, Japan) coupled with tandem MS equipment was employed. The data obtained from the LC-ESI-MS/MS analysis were processed using Shimadzu Lab Solutions software.

#### 4.4.2. Quantification of Marker Bioactive Compounds by HPTLC

The qualitative and quantitative analysis of PCA in the fractions was conducted using HPTLC, as outlined by Bardakcı et al. [82]. The procedure used 20 cm × 10 cm glass plates coated with HPTLC silica gel 60 F254 (Merck, Darmstadt, Germany). The mobile phase was a mixture of EtOAc, CHCl_3_, FA, AA, and H_2_O in the ratio 100:25:10:10:11. The sample solutions were prepared at 1 mg/mL, while he standards were at 100 µg/mL in methanol, with each solution filtered through a 0.45 µm syringe filter. The extracts (2–40 µL) and standards (2–12 µL) were applied to the silica plates as 6 mm bands using the CAMAG Automatic TLC Sampler IV. Development occurred in the CAMAG Automatic Development Chamber-2 (ADC-2), which was saturated and pre-conditioned with MgCl_2_ at 33% RH for 10 min. After derivatization with Natural Product Reagent (NPR), densitometric analysis was carried out with a CAMAG TLC Scanner IV in fluorescence mode, using a slit size of 5 × 0.2 mm and a scanning speed of 20 mm/s. The standard content was determined by comparing the area under the curve (AUC) at 366 nm to a calibration curve. VisionCATS software was used for device control and analysis, with correlation coefficients (r^2^) greater than 0.98.

### 4.5. Development of Emulgel Formulation

Emulgel formulations were chosen as the carriers for the primary methanol extracts of the plants. Details of the emulsion components are provided in Table 11.

To prepare the extract-loaded emulgel formulation, the extracts were mixed into the oil phase. Both the oil and water phases were heated to 50 °C. The oil phase was then added gradually to the water phase while stirring vigorously. The mixture continued to be stirred at high speed until it cooled. To enhance the stability of the emulsion, the hydrophilic–lipophilic balance (HLB) was adjusted to between 11 and 12. Once the emulsion had cooled, the gel-forming agent was incorporated.

For the gel formulations, 2% Carbopol 934 was added to the emulsion, followed by a few drops of triethanolamine to complete the emulgel formulation.

#### 4.5.1. Characterization Studies of the Formulation

##### pH Measurement

A Mettler Toledo-USA pH meter (Greifensee, Switzerland) device was used for the pH measurements of the formulations. First, 1 g of the formulation was dispersed in 25 mL of distilled water. Then, a pH meter probe was dipped into it and the reading was performed. The measurements were carried out at room temperature and repeated 3 times [83].

##### Viscosity Measurement

A Brookfield DVE Viscosimeter (London, UK) was used to measure the viscosities of the formulations. For measurements, spindle number 07 was rotated at 100 rpm at room temperature until the measurement value became constant. The measurement was repeated 3 times.

##### Determination of Content Quantity in the Formulations

Table 1 shows that protocatechuic acid was the highest in terms of quantity. For this reason, protocatechuic acid was preferred as a reference agent. In the content quantity analysis, 1 g of the formulation was taken and distributed in 5 mL of methanol. The lid was tightly closed and wrapped with parafilm to prevent evaporation. It was then centrifuged at 5000 rpm for 30 min. The supernatant was taken and analyzed by the HPLC method.

For the HPLC analysis, a SHIMADZU LC 2030C 3D Nexera-i Plus (Kyoto, Japan) device was studied with a C18 column (GL Science, Tokyo, Japan −5 μm, 4.6 × 250 mm) at 30 °C at 260 nm. The flow rate used was 1 mL/min, and the injection volume was determined as 20 μL. The mobile phase was prepared as 0.2 M sodium acetate trihydrate/methanol (90:10) with pH 3.6. The chromatographic conditions were optimized and maintained at a constant throughout the experiment. The method was partially validated according to ICH guidelines with respect to the system suitability, linearity, limit of detection (LOD) and limit of quantitation (LOQ), precision, accuracy, specificity, and selectivity.

##### Spreadability and Texture Profile Analysis

A TA-XT Plus texture analyzer (Stable Micro Systems, Surrey, UK), operating in TPA mode, was employed to evaluate the texture profiles and spreadability. To assess the texture profiles, 10 g of the formulation in 50 mL falcon tubes was centrifuged at 4000 rpm for 10 min using an Eppendorf 5810R centrifuge (New York, NY, USA). The sample was placed 10 mm above the probe, which was lowered into the sample by 10 mm at a constant speed of 1 mm·s^−1^ for 2 min, controlled by the trigger force. The probe was then retracted at a speed of 0.5 mm·s^−1^, and after a 5 s interval, a second compression was performed. These analyses were repeated three times at a temperature of 25 °C. In the spreadability test, 2 g of the formulation was positioned inside the female cone, and the male cone advanced towards it to a distance of 23 mm, with a test speed of 3 mm/s and a post-test speed of 10 mm/s. The gel’s spreadability was measured in terms of firmness (maximum positive force) and stickiness (maximum negative force) [38].

#### 4.5.2. In Vitro Release Studies

The in vitro release properties of the suspended herbal extract in distilled water (ETS) and ETF formulation were assessed using PBS/methanol (70:30) to achieve sink conditions at 100 rpm. The temperature was regulated at 32 ± 0.5 °C to replicate cutaneous temperature. Two grams of the formulation and suspended herbal extract were placed onto the dialysis membrane (Spectra/Por 2, 12–14 kD MWCO), which was thereafter sealed and submerged in the release medium. At designated time intervals, 0.5 mL of the medium was withdrawn, and an equal volume of new medium was added, therefore establishing sink conditions. The MSM release and MSM content were assessed utilizing the HPLC method [83].

##### Kinetic Release Modeling

In order to determine the most probable method of protocatechuic acid release, a number of mathematical models were used to analyze the results from the in vitro MSM release studies [84]. The formulations with the highest regression (r^2^) values had their correlation coefficients employed to choose the optimal model. The release mechanism was examined utilizing zero-order, first-order, Higuchi, and Hixson–Crowell kinetic models. Additionally, the Korsmeyer–Peppas equation was employed for kinetic analysis.

##### Similarity Studies

A novel mathematical approach developed by Moore and Flanner was utilized for the statistical analysis of in vitro release patterns of the formulations. This model-independent approach utilizes a difference factor (*f*1) and a similarity factor (*f*2) to assess the dissolution profiles. F1 values up to 15 and F2 values between 50 and 100 indicate that the release profiles are comparable [84]. Statistical analysis was performed for the intraformulation comparisons.

Using Equations (1) and (2), the difference factor (*f*1) and the similarity factor (*f*2) were determined.

(1) f1=∑t=1nRt−Tt∑t=1nRt×100

(2) $$f2=50xlog\left[{\left(1+\left(\frac{1}{n}\right){\sum \left(Rt-Tt\right)}^{2}\right)}^{-0.5}\times 100\right]$$

where *n* represents the number of sampling time points, and *R_t_* and *T_t_* denote the percentage of drug released from the reference and test formulations at time *t*, respectively. The difference factor (*f*_1_) quantifies the percent deviation between the two release profiles at each time point, reflecting their dissimilarity. The similarity factor (*f*_2_), derived from a logarithmic transformation of the squared differences, indicates the degree of agreement between the profiles. Interpretation of these factors is based on established, though arbitrary, thresholds in the literature [85].

##### Ex Vivo Permeation and Penetration Studies

The Franz diffusion cell was utilized to perform ex vivo permeation and penetration investigations on the formulations. First, 9 mL of a 30:70 methanol/PBS medium was utilized to fill the cells, and the temperature was regulated at 32 ± 0.5 °C while stirring at 50 rpm to provide sink conditions. Parafilm was employed to inhibit the evaporation of the 1 g ETF and ETS within the sealed donor compartment. A 0.5 mL aliquot of the receiver medium was obtained and immediately replaced with an equivalent volume of medium to maintain a final volume. Mice dorsal skin was used for the permeation/penetration studies. The experiment was performed thrice. The samples were tested using HPLC. The below calculation was used to determine the total amount that was able to pass through the skin.

Equation (3) shows the calculation of the accumulated amount of protocatechuic acid (reference agent) at a given time t:

(3) $$Qn=\frac{\left(Cn+\frac{V}{V0}\sum_{i=1}^{n-1}Ci\right)V0}{S}$$

S stands for the effective diffusion area (S  =  0.95 cm^2^), Ci is the sample MSM concentration, V0 and Vi are the volumes of the receiver solution and sample, respectively, and Qn is the total amount accumulated at a specific time t. Cn is the MSM concentration in the receiver solution at each sampling interval. As a function of time (t), the cumulative quantity of protocatechuic acid penetrated (Qn) was displayed for each formulation. To determine the steady-state penetration rate of protocatechuic acid through the cornea (Jss, g/cm^2^/h), the gradient of the linear section of the Qn versus time plots was used. Kp, in cm/h, is the permeability coefficient calculated using the following formula [59].

Equation (4) shows the calculation of the permeability coefficient.

(4) $$Kp=\frac{Jss}{C0}$$

The constant MSM concentration in the vehicle is denoted by C0, the steady-state flow is represented by Jss, and the permeability coefficient is Kp. After 24 h of contact, each skin was cleaned in order to conduct a penetration test. PCA skin retention was assessed following 24 h of exposure to the formulation. After the contact period, the skin surface was carefully cleansed to remove any residual formulation and ensure that only retained PCA was measured. Subsequently, the skin samples were excised and homogenized by cutting them into small pieces to facilitate extraction.

To extract the retained PCA, the skin tissues were treated with ethanol and mixed thoroughly until a homogeneous solution was obtained. This ethanolic extract was then analyzed using a validated high-performance liquid chromatography (HPLC) method to quantify the amount of PCA retained within the skin layers.

### 4.6. In Vitro Investigation of Antioxidant Potential

Antioxidant activity was evaluated using CUPRAC, TOAC, FRAP, and DPPH radical scavenging assays. The DPPH assay measures the reduction in DPPH radicals by observing a decrease in absorbance at 517 nm after a 30 min incubation in darkness. For the FRAP assay, the reduction in Fe(III) to Fe(II) forms a colored complex, and absorbance is measured at 593 nm after a 30 min incubation at 37 °C. The CUPRAC and TOAC assays assessed the reduction potential of the samples for cupric and Mo (VI) ions, respectively, with ascorbic acid as the reference. All the assays were performed in triplicate, with the results expressed as mean values ± standard deviation [86,87].

### 4.7. Skin Enzyme Inhibition Assays

#### 4.7.1. Anticollagenase Activity

A 50 mM Tricine buffer solution at pH 7.5 was prepared, containing 400 mM NaCl and 10 mM CaCl_2_. Collagenase enzyme from *Clostridium histolyticum* was dissolved in this buffer to achieve an initial concentration of 0.8 units/mL. N-[3-(2-furyl)-acryloyl]-Leu-Gly-Pro-Ala (FALGPA) was dissolved in the Tricine buffer at a concentration of 2 mM to serve as the substrate. The extracts were incubated with the collagenase enzyme in the buffer for 15 min before the substrate was added to start the reaction. The final reaction mixture, with a total volume of 150 μL, included 0.8 mM FALGPA, 0.1 unit ChC, and 25 μg of the test extract. Negative controls used only the Tricine buffer, and absorbance was measured immediately after the substrate addition. EGCG was used as a positive control [88].

#### 4.7.2. Antielastase Activity

To evaluate the antielastase activity of the extracts, a 0.2 mM tris-HCl buffer (pH 8.0) and a stock solution of elastase enzyme (3.33 mg/mL) prepared in sterile water were utilized. The substrate, N-succinyl-Ala-Ala-p-nitroanilide (AAAPVN), was dissolved in the buffer at a concentration of 1.6 mM. The plant extracts were pre-incubated with the enzyme for 15 min before adding the substrate. Epigallocatechin gallate (EGCG) was used as a positive control, while methanol acted as the negative control. Following substrate addition, the mixture was incubated for another 15 min, and absorbance changes were measured using a Thermo Scientific (Waltham, MA, USA) Multiskan SkyHigh Microplate Spectrophotometer [89].

#### 4.7.3. Antihyaluronidase Activity

To evaluate the antihyaluronidase activities of the extracts, 50 mM (pH: 7) Tris-HCL buffer solution and 10% concentration of cetylpyridinium chloride solution were prepared (*w*/*v*). Hyaluronic acid substrate at a concentration of 0.4 mg/mL and 800 U/mL hyaluronidase enzyme were dissolved in distilled water. The final volume was 110 μL and contained 10 μL substrate, 10 μL enzyme, 10 μL plant extract, and 70 μL Tris-HCL buffer solution. First, 10 μL of the plant extract was incubated at 37 °C with 10 μL of enzyme and 70 μL of buffer solution for 1 h. Then, 10 μL of cetylpyridinium chloride was added and incubated for another 1 h at 37 °C. Then, 10 μL of substrate was added and the absorbance values were read. Negative controls were made with distilled water. In addition, 1.1 mg/mL tannic acid was used as a reference. The absorbances were read at 415 nm [41].

#### 4.7.4. Antityrosinase Activity

In vitro antityrosinase activity was assessed using a modified method based on Ersoy et al. A 100 mM phosphate buffer with a pH of 6.8 was prepared, and L-DOPA was used as the substrate. Initially, 100 μL of the phosphate buffer, 20 μL of a 1 mg/mL plant extract, and 20 μL of tyrosinase enzyme were incubated at 37 °C for 10 min. Next, 20 μL of 3 mM DOPA was added, and the mixture was incubated again at 37 °C for 30 min. The absorbance was measured at 492 nm, with kojic acid used as the reference standard [89].

### 4.8. Stability Studies

ETF underwent a 12-month evaluation in stability cabinets at 5 ± 3 °C (refrigerated), 25 ± 2 °C with 60% ± 5% relative humidity, and 30 ± 2 °C with 65 ± 5% relative humidity, in compliance with ICH guidelines [90]. Viscosity, pH, and content quantification was performed at t = 0 and after the 12th month.

### 4.9. Statistical Analysis

The experiments presented in the article were carried out by repeating at least three times in independent time sands. All the experiments were performed with GraphPad Prism software version 8.

## Figures and Tables

**Table 1:** LC-MS/MS results of ETM extract.

	Analytes	R.T. ^a^	M.I.s (*m*/*z*) ^b^	F.I.s (*m*/*z*) ^c^	Ion. Mode	Quantification (μg/g)
1	Quinic acid	3.0	190.8	93.0	Neg	1.086
2	Fumaric aid	3.9	115.2	40.9	Neg	0.963
3	Equisetic acid	4.0	172.8	129.0	Neg	7.865
4	Gallic acid	4.4	168.8	79.0	Neg	0.091
5	Epigallocatechin	6.7	304.8	219.0	Neg	-----
6	Protocatechuic acid	6.8	152.8	108.0	Neg	8.503
7	Catechin	7.4	288.8	203.1	Neg	-----
8	Gentisic acid	8.3	152.8	109.0	Neg	-----
9	Chlorogenic acid	8.4	353.0	85.0	Neg	0.013
10	Protocatechuic aldehyde	8.5	137.2	92.0	Neg	2.607
11	Tannic acid	9.2	182.8	78.0	Neg	0.199
12	Epigallocatechin gallate	9.4	457.0	305.1	Neg	-----
13	Cynarin	9.8	515.0	191.0	Neg	-----
14	4-OH Benzoic acid	10.5	137,2	65.0	Neg	0.602
15	Epicatechin	11.6	289.0	203.0	Neg	-----
16	Vanilic acid	11.8	166.8	108.0	Neg	-----
17	Caffeic acid	12.1	179.0	134.0	Neg	0.149
18	Syringic acid	12.6	196.8	166.9	Neg	-----
19	Vanillin	13.9	153.1	125.0	Poz	-----
20	Syringic aldehyde	14.6	181.0	151.1	Neg	-----
21	Daidzin	15.2	417.1	199.0	Poz	-----
22	Epicatechin gallate	15.5	441.0	289.0	Neg	-----
23	Piceid	17.2	391.0	135/106.9	Poz	-----
24	p-Coumaric acid	17.8	163.0	93.0	Neg	-----
25	Ferulic acid-D3-IS ^d^	18.8	196.2	152.1	Neg	N.A.
26	Ferulic acid	18.8	192.8	149.0	Neg	-----
27	Sinapic acid	18.9	222.8	193.0	Neg	-----
28	Coumarin	20.9	146.9	103.1	Poz	0.034
29	Salicylic acid	21.8	137.2	65.0	Neg	0.028
30	Luteolin 7-O-glucoside	23.7	447.0	284.0	Neg	-----
31	Miquelianin	24.1	477.0	150.9	Neg	-----
32	Rutin-D3-IS	25.5	612.2	304.1	Neg	N.A.
33	Rutin	25.6	608.9	301.0	Neg	0.076
34	Isoquercetin	25.6	463.0	271.0	Neg	0.097
35	Hesperidin	25.8	611.2	449.0	Poz	0.025
36	o-Coumaric acid	26.1	162.8	93.0	Neg	-----
37	Genistin	26.3	431.0	239.0	Neg	-----
38	Rosmarinic acid	26.6	359.0	197.0	Neg	-----
39	Ellagic acid	27.6	301.0	284.0	Neg	-----
40	Apigenin 7-glucoside	28.2	431.0	269.0	Neg	0.035
41	Quercitrin	29.8	447.0	301.0	Neg	-----
42	Astragalin	30.4	447.0	255.0	Neg	4.764
43	Nicotiflorin	30.6	592.9	255.0/284.0	Neg	5.334
44	Fisetin	30.6	285.0	163.0	Neg	-----
45	Daidzein	34.0	253.0	223.0	Neg	-----
46	Quercetin-D3-IS	35.6	304.0	275.9	Neg	N.A.
47	Quercetin	35.7	301.0	272.9	Neg	0.059
48	Naringenin	35.9	270.9	119.0	Neg	0.214
49	Hesperetin	36.7	301.0	136.0/286.0	Neg	-----
50	Luteolin	36.7	284.8	151.0/175.0	Neg	0.012
51	Genistein	36.9	269.0	135.0	Neg	-----
52	Kaempferol	37.9	285.0	239.0	Neg	0.861
53	Apigenin	38.2	268.8	151.0/149.0	Neg	0.045
54	Amentoflavone	39.7	537.0	417.0	Neg	0.005
55	Chrysin	40.5	252.8	145.0/119.0	Neg	0.177
56	Acacetin	40.7	283.0	239.0	Neg	0.038

^a^ R.T.: Retention time, ^b^ M.I.s (*m*/*z*): Molecular ions of the standard analytes (*m*/*z* ratio), ^c^ F.I. (*m*/*z*): Fragment ions. ^d^ Internal standard. N.A.: Not applicable.

**Table 2:** Quantification of the major bioactive compounds by HPTLC.

Compound	ETM	ETH	ETC	ETE	ETB	ETW
Protocatechuic acid	8.49 ± 0.15	nd	nd	137.4 ± 1.99	31.95 ± 1.44	nd

Quantification data of protocatechuic acid as μg/g in all fractions by HPTLC analysis. nd: non-detected. ETM: methanolic extract of ET, ETH: hexane fraction of ET, ETC: chloroform fraction of ET, ETE: ethyl acetate fraction of ET, ETB: *n*-butanol fraction of ET, ETW: remaining water fraction of ET.

**Table 3:** Characterization results of blank and loaded formulations.

Formulation/Characterization	F *	ETF
pH	5.4 ± 0.1	6.5 ± 0.1
Viscosity (P)	24.227 ± 0.228	6.440 ± 0.080
Drug Content (%)	-	77.19 ± 2.07

F: blank gel, ETF: emulgel loaded with methanolic extract of *Equisetum telmateia* (ETM).* Blank gel was used in a previous study given in the references [41].

**Table 4:** Texture profile analysis results of blank and methanol extract-loaded formulations, including hardness, adhesiveness, elasticity, and cohesiveness (mean ± SD).

Formulation	Hardness (g) ± SS	Adhesiveness (g·s) ± SS	Elasticity ± SS	Cohesiveness ± SS
F	−3.345 ± 0.423	−90.678 ± 0.391	0.235 ± 0.053	0.855 ± 0.139
ETF	−3.699 ± 0.273	−58.000 ± 1.665	0.143 ± 0.004	0.903 ± 0.058

**Table 5:** Spreadability results of blank and results of blank and methanol extract-loaded formulations, including firmness, work of shear, stickiness, and work of adhesion (mean ± SD).

Formulation	Firmness (g)	Work of Shear (g·s)	Stickiness (g)	Work of Adhesion (g·s)
F	1591.67 ± 6.01	1657.73 ± 32.20	−1287.72 ± 14.37	−214.75 ± 12.33
ETF	1205.36 ± 2.93	1078.68 ± 15.75	−964.46 ± 10.19	−257.98 ± 19.48

**Table 6:** In vitro release kinetics results of ETS and ETF.

Formulations	ETS			ETF		
Models	r^2^	m	n	r^2^	m	n
Zero-order	0.305	1.4186	80.407	0.748	4.1964	26.354
First-order	0.278	0.0079	1.8905	0.676	0.0344	1.4209
Hixson–Crowell	0.288	−0.0257	−0.293	0.713	−0.0969	0.9114
Higuchi	0.510	10.239	66.187	0.865	25.194	−3.662
Korsmeyer–Peppas	0.840		2.4873	0.992		2.0815

**Table 7:** The calculated difference (*f*_1_) and similarity (*f*_2_) factor for ETS and ETF.

Method of Release	Reference Formulation	Experimental Formulation	*f*_1_	*f*_2_
Dialysis bag	ETS	ETF	66	15
	ETF	ETS	193	15

**Table 8:** In vitro tests for the antioxidant potential of different extracts of ET and ETF.

	ETM *	ETH *	ETC *	ETE *	ETB *	ETW *	ETF
DPPH ^1^	2639 ± 9 ^a^	1812 ± 20 ^b^	1949 ± 42 ^c^	2654 ± 4 ^a^	2684 ± 7 ^a^	2570 ± 23 ^d^	1124 ± 27 ^e^
FRAP ^2^	1.37 ± 0.06 ^a^	0.67 ± 0.02 ^b^	0.60 ± 0.29 ^b^	2.40 ± 0.18 ^c^	3.02 ± 0.40 ^d^	1.02 ± 0.09 ^ab^	1.17 ± 0.16 ^ab^
CUPRAC ^3^	214.64 ± 10.73 ^a^	122.75 ± 9.95 ^b^	161.42 ± 8.66 ^cf^	428.88 ± 12.66 ^d^	321.28 ± 0.38 ^e^	147.03 ± 8.60 ^bc^	184.31 ± 2.91 ^f^
TOAC ^3^	158.09 ± 9.81 ^a^	173.60 ± 8.77 ^a^	218.22 ± 3.41 ^b^	316.91 ± 13.89 ^c^	265.60 ± 8.39 ^d^	157.07 ± 10.84 ^a^	173.41 ± 2.23 ^a^

^1^ Results were expressed as the mean of triplicates ± standard deviation (S.D.) and as mg butylated hydroxyl toluene equivalents (BHTEs) in 1 g sample. ^2^ Results were expressed as the mean of triplicates ± standard deviation (S.D.) and as mM FeSO_4_ equivalents in 1 g sample. ^3^ Results were expressed as the mean of triplicates ± standard deviation (S.D.) and as mg ascorbic acid equivalents (AAE) in 1 g sample. * ETM: methanolic extract of ET, ETH: hexane fraction of ET, ETC: chloroform fraction of ET, ETE: ethyl acetate fraction of ET, ETB: n-butanol fraction of ET, ETW: remaining water fraction of ETW, ETF: Emulgel formulation loaded with methanol extract of ET. different letters in the same row indicate significance (*p* < 0.05).

**Table 9:** Characteristics results of penetration and permeation studies of ETS and ETF.

Formulation Code	Jss (mg·cm^−2^·h^−1^)	K_p_ × 10^−4^ (cm/h)	Q_n_ (mg·cm^−2^)	Skin Retention (mg·cm^−2^)
ETS	1.1040 ± 0.1520	347.0 ± 48.0	20.17 ± 3.66	1.28 ± 0.47
ETF	0.1502 ± 0.0392	47.0 ± 14.0	5.16 ± 3.74	3.06 ± 0.21

**Table 10:** Stability results of ETF.

4 ± 2 °C			
Time (Month)	Content Quantification (%)	pH	Viscosity (P)
0	100.00 ± 2.68	6.50 ± 0.10	6.440 ± 0.080
12	92.63 ± 8.44	6.33 ± 0.01	6.493 ± 23.09
25 ± 2 °C			
Time (Month)	Content Quantification (%)	pH	Viscosity (P)
0	100.00 ± 2.68	6.50 ± 0.10	6.440 ± 0.080
12	79.53 ± 8.73	6.31 ± 0.01	6.466 ± 0.023
40 ± 2 °C			
Time (Month)	Content Quantification (%)	pH	Viscosity (P)
0	100.00 ± 2.68	6.50 ± 0.10	6.440 ± 0.080
12	72.14 ± 0.66	6.26 ± 0.02	6.400 ± 0.040

**Table 11:** Components and amounts of excipients of the emulgel formulation.

Ingredients	Amount (%)
**Oil Phase**	
Oleic acid	5
Span 80	1.37
Methyl paraben	0.1
**Water Phase**	
Glycerine	1.8
Ethyl alcohol	3
Tween 60	3.63
Propyl paraben	0.1
Distilled water	q.s. 100
Carbopol 934	2
Plant extract	4
Triethanolamine	q.s.

## Data Availability

The original contributions presented in this study are included in the articlematerial. Further inquiries can be directed to the corresponding author(s).
